# Antioxidant and laxative effects of taurine-xylose, a synthetic taurine-carbohydrate derivative, in loperamide-induced constipation in Sprague-Dawley rats

**DOI:** 10.20463/jenb.2019.0025

**Published:** 2019-12-31

**Authors:** Hee Geun Jo, Min Ji Kim, Bo Yeong Moon, Sun Hee Cheong

**Affiliations:** 1 Department of Marine Bio-Food Sciences, Chonnam National University, Yeosu Republic of Korea

**Keywords:** Antioxidant enzyme activities, Constipation, Laxative effects, Loperamide, Taurine-xylose

## Abstract

**[Purpose]:**

In this study, we examined the in vitro antioxidant activities and laxative effects of taurine-xylose (T-X), a synthetic taurine-carbohydrate derivative, in a rat model of constipation induced by loperamide.

**[Methods]:**

The animals were divided into four treatment groups: normal untreated rats (NOR group), loperamide-treated control rats (CON group), loperamide and taurine-xylose (15 mg/kg)-treated rats (T-X group), and loperamide and commercial Dulcolax S (5.5 mg/kg)-treated rats (DS group).

**[Results]:**

In the present study, T-X exhibited potent reducing power and free radical scavenging activities for DPPH (1,1-diphenyl-2-picrylhydrazyl) and ABTS+ (2,2′-azino-bis (3-ethylbenzothiazoline 6-sulfonic acid ammonium salt) radicals. The laxative effects of T-X were dependent on food, body weight, fecal properties, gastrointestinal transit (GIT) ratio, and serum metabolic parameters. In the T-X group, the number, wet weight, and water content of fecal pellets were noticeably increased compared to those in the loperamide-induced group. T-X treatment significantly increased the activities of hepatic antioxidant enzymes, including those of glutathione peroxidase (GSH-Px), superoxide dismutase (SOD), and catalase (CAT), relative to those in loperamide-induced constipated rats. Furthermore, the GIT ratio and loperamide-induced metabolic parameters in serum, including gastrin (GAS), motilin (MTL), and somatostatin (SS) levels, were significantly improved by T-X treatment.

**[Conclusion]:**

These results suggest that taurine-xylose exerts antioxidant activities and laxative effects on loperamide-induced constipation by promoting gastrointestinal motility.

## INTRODUCTION

Recently, owing to rapid improvements in the standard of living and dietary habits, the incidence of metabolic disease and constipation in people has increased^1^. Constipation is said to have many deleterious outcomes, including skin aging, headaches, pimples, hemorrhoids, and colorectal cancer^2,3^. Constipation is commonly characterized by infrequent stools and difficulty passing stools, or both, with movement once every 3-4 days or less^4-6^. Treating and preventing constipation has emerged as a contemporary challenge. There are many drugs for constipation available in the market. However, these drugs have temporary effects and may have adverse reactions such as abdominal distension or diarrhea^7,8^. Indeed, research has focused on finding drugs with the same properties as those of commercially available drugs but without side effects.

Loperamide is frequently used as a constipation-inducing drug^9^. The inhibition of intestinal secretion and peristaltic activity induced by loperamide, however, can have harmful effects on antioxidant enzyme activity. Further, disturbance in intestinal absorption or secretion can cause oxidative stress in the intestine^10,11^. Therefore, it is necessary to identify and study foods and natural products that are effective in preventing and treating constipation.

Taurine is a sulfur-containing amino acid that is not used for protein synthesis or as an energy source. It is most abundant in the brain, heart, eyeball, muscle tissue, and liver^12,13^. It also has important roles in neuroprotection, cell membrane stabilization, detoxification, antioxidation, osmotic pressure control, diabetic complication reduction, atherosclerosis, and gastrointestinal damage^14-16^. Although these beneficial effects of taurine have been investigated, antioxidant activities and laxative effects of T-X, a synthetic taurine-carbohydrate derivative, remain unclear. In this study, we investigated the antioxidant and laxative effects of T-X on loperamide-induced constipation in rats. This is the first report of such effects.

## METHODS

### Materials

Synthesized T-X was donated by Prof. Sung Hoon Kim (Kunkuk University, South Korea). 1,1-Diphenyl-2-picrylhydrazyl (DPPH), 2,2'-azino-bis (3-ethylbenzothiazoline 6-sulfonic acid ammonium salt) (ABTS), potassium persulfate, sodium phosphate buffer, potassium ferricyanide, trichloroacetic acid (TCA), ferric chloride, carmine, and loperamide hydrochloride were purchased from Sigma-Aldrich Chemical Co. (St. Louis, MO). All reagents used were of analytical grade.

### DPPH radical scavenging activities

The DPPH assay was performed with some modifications according to the method of Brand-Williams et al.^17^. T-X (100 μL at 3.125, 6.25, 12.5, 25, 50, 100, 200, and 400 mg/mL) was added to 200 μL of 0.2 mM DPPH solution dissolved in methanol. The samples were incubated for 30 min at room temperature in the dark, and then absorbances at 517 nm were measured using a microplate reader (Multiskan FC 357, Thermo Scientific, China).

### ABTS radical scavenging activities

The ABTS assay was performed with some modifications according to the method of Re et al.^18^. Briefly, ABTS radical cations were prepared by mixing 7.4 mM ABTS solution and 2.6 mM potassium persulfate solution in equal quantities and allowing the mixture to react for 24 h at room temperature in the dark before use. The ABTS+ solution was diluted with deionized water to obtain an absorbance at 734 nm. T-X (50 μL at 3.125, 6.25, 12.5, 25, and 50 mg/mL) was added to 950 μL of diluted ABTS+ solution. The samples were incubated for 30 min at room temperature in the dark, and then absorbances were measured at 734 nm using a microplate reader.

### Measurement of reducing power

The reducing power assay was used with some modifications according to the method of Oyaizu^19^. T-X (100 μL at 3.125, 6.25, 12.5, 25, and 50 mg/mL) was added to 0.2 M sodium phosphate buffer (pH 6.6) and 10% potassium ferricyanide were mixed and added in an amount equal to that of the sample, and the mixture was allowed to react for 20 min at 50°C. The resulting solution was mixed with 100 μL of 10% trichloroacetic acid and centrifuged at 1,000 × g for 10 min. A 200-μL sample of the supernatant was mixed with 40 μL of 0.1% ferric chloride, and then absorbances were measured at 700 nm using a microplate reader.

### Animals

Twenty-four male Sprague-Dawley rats (5 weeks old) were purchased from Samtacho (Osan, Korea) and individually housed in a room with temperature (23 ± 2°C) and humidity (55 ± 10%) controlled under 12 h light/dark cycle. All rats consumed a standard irradiated chow diet (Purina Mills, Seoungnam, Korea) and water *ad libitum* to stabilize their metabolic condition for 1 week. The animal procedures were conducted in accordance with the Guidelines for Care and Use of Laboratory Animals and were approved by the Animal Ethnics Commit-tee of Chonnam National University, Yeosu, Korea (approval number: CNU IACUC-YS-2016-7).

### Induction of constipation

After 1 week of adaptation, the rats were randomly divided into four treatment groups (n = 6 each): normal untreated rats (NOR group), loperamide-treated control rats (CON group), loperamide and T-X (15 mg/kg)-treated rats (T-X group), and loperamide and commercial Dulcolax S (5.5 mg/kg)-treated rats (DS group). All rats were provided AIN-76A basal diets and water *ad libitum*. Constipation was induced in the rats by oral administration of 1 mL loperamide (4 mg/kg) suspended in 0.9% sodium chloride twice per day, at 9 am and 6 pm, for 14 days. The control group was administered normal saline only. During the experimental periods, T-X (15 mg/kg) was orally administered to rats at 10 am. A comparison group was treated with commercial Dulcolax S (5.5 mg/kg) as the standard drug. We measured daily food intake, water intake, and body weight gain of all the rats, and treatment continued for 14 days. The feed efficiency ratio (FER) in the experimental period was calculated by dividing the dietary intake quantities by body weight gain.

### Determination of number, weight, and water content of fecal pellets

The number and weight of fecal pellets from individual rats were determined daily at 9 am during the experimental period. We dried fecal pellets for 24 h at 70°C and determined the water content using the following equation: Fecal water content (%) = [(fecal wet weight - fecal dry weight)/fecal wet weight] × 100.

### Biochemical analysis of blood

Blood was collected from the heart using a syringe under ether anesthesia. The serum was separated by centrifugation at 2,000 × g for 20 min and stored at -80°C. Organs, namely liver, heart, and kidneys, were removed, weighed, frozen in liquid nitrogen, and stored at -80°C until the biochemical analysis was conducted. Serum total protein, albumin, aspartate aminotransferase (AST), alanine transaminase (ALT), blood urea nitrogen (BUN), and creatinine concentrations were determined using an automatic biochemical analyzer (Hitachi 747; Hitachi Co., Tokyo, Japan).

### Total glutathione (GSH) content and hepatic antioxidant enzyme activities

Liver tissue was homogenized in a glass-Teflon homogenizer with 50 mM phosphate buffer (pH 7.4) to obtain 1:9 (w/v) whole homogenate that was then centrifuged at 25,000 rpm for 20 min at 4°C. The supernatant was used to assess hepatic antioxidant enzyme activities. Total GSH content and superoxide dismutase (SOD) and catalase (CAT) activities were determined using a commercially available kit supplied by Biovision (CA, USA) according to the methods suggested by the manufacturer. Hepatic glutathione S-transferase (GST) activity was determined using 1-chloro-2,4-dinitrobenzene (CDNB) in the presence of 0.1 mM GSH following the method of Habig et al.^20^. The formation of dinitrophenyl thioether in response to GST was monitored for 3 min at 37°C using a spectrophotometer at 340 nm. Glutathione reductase (GR) was measured using the method of Pinto et al. [21]. Briefly, the supernatant was mixed with 5 mM NADPH and 1 M glutathione disulfide (GSSG), and the formation of NADP+ was monitored with a spectrophotometer at 340 nm. For glutathione peroxidase (GPx) activity, the supernatant was mixed with 100 mM GSH, 1 mM EDTA, 5 mM NADPH, and 1 unit of GR in 0.1 M phosphate buffer (pH 7.0), and then incubated for 3 min. 10 mM cumene hydroperoxide was added to the reaction mixture, and the oxidation of NADPH into NADP+ was determined using a spectrophotometer at 340 nm.

### Measurement of the GIT ratio

The GIT ratio was determined using the method of Niwa et al. with some modifications^22^. On the 15th day of the experimental period, 1 mL carmine (3 g suspended in 50 mL of 0.5% carboxymethylcellulose) was orally administered to the rats as a marker. After 30 min, the animals were sacrificed, and then small intestines were quickly removed. We determined the distance over which carmine had travelled and the total length of the small intestine to calculate the GIT ratio. The GIT ratio was expressed as the percentage of distance travelled by carmine relative to the total length of the small intestine.

### Measurement of serum gastrin (GAS), motilin (MTL), somatostatin (SS), and calcitonin gene-related peptide (CGRP) concentrations

The concentrations of GAS, MTL, SS, and CGRP in serum were evaluated using commercially available ELISA kits.

### Statistical analysis

All data are expressed as the mean ± SD. Statistical analyses were performed using the IBM SPSS Statistics version 20.0 (Chicago, IL, USA). Multiple groups were compared by one-way analysis of variance, followed by Tukey-Kramer multiple range testing to determine significant differences in all parameters. Values were considered statistically significant at P < 0.05.

## RESULTS

### Free radical scavenging activities of T-X

**Figure 1.**
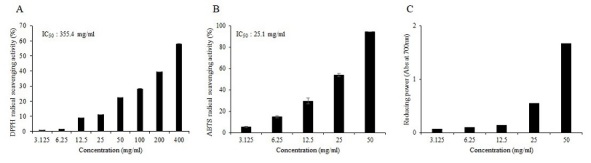
Free radical scavenging activities of T-X. (A) DPPH radical scavenging activity, (B) ABTS radical scavenging activity, and (C) reducing power. IC50 values are expressed as milligrams of T-X per milliliter organic solvent.

As shown in Fig. 1, we investigated the in vitro antioxidant activities of T-X using DPPH, ABTS, and reducing power assays. The IC_50_ value, the concentration inhibiting 50% of free radical production efficacy of T-X against DPPH radicals, was 355.4 mg/mL. In addition, T-X exhibited stronger scavenging activity (25.1 mg/mL) against ABTS radicals than the other treatments, and absorbances in the reducing power assay tended to increase as the concentration of T-X increased. These results showed that T-X effectively scavenged various reactive radicals in a concentration-dependent manner.

### Effects of T-X on body weight, food intake, and FER

The effects of T-X on body weight, food intake, and FER in rats are presented in Table 1. Final body weight, body weight gain, and food intake were significantly higher in the NOR group than in the other groups. Food intake tended to be decreased in the CON group compared to that in the T-X and DS groups. In addition, there was no significant difference in FER among the groups.

**Table 1.** Effects of T-X on body weight gain, food intake, and food efficiency ratio in loperamide-induced constipated rats

	Groups			
	NOR	CON	T-X	DS
Initial body weight (g)	77.3 ± 1.5^NS^	77.0 ± 4.4	76.6 ± 2.5	76.7 ± 1.2
Final body weight (g)	215.3 ± 4.9^a^	187.3 ± 12.0^b^	178.6 ± 9.0^b^	183.7 ± 9.3^b^
Body weight gain (g)	138.0 ± 5.3^a^	110.3 ± 13.4^b^	102.0 ± 10.7^b^	107.0 ± 8.9^b^
Food intake (g/day)	18.8 ± 0.5^a^	14.9 ± 0.4^b^	15.2 ± 0.5^b^	15.4 ± 0.8^b^
FER^1)^	7.30 ± 0.08^NS^	6.95 ± 0.88	6.97 ± 0.69	7.12 ± 0.18

NOR: normal diet group, CON: loperamide-induced constipation group (4 mg/kg, p.o.), T-X: taurine-xylose (15 mg/kg, p.o.) and loperamide-treated group, DS: Dulcolax S (5.5 mg/kg, p.o.) and loperamide-treated group. Values are the mean ± SD (n = 6). NS: not significantly different among groups. Values not sharing a common letter are significantly different at P < 0.05 by the Tukey-Kramer multiple comparison test. 1)FER: food efficiency ratio = body weight gain (g)/food intake (g).

### Effect of T-X on organ weights in the loperamide-induced constipated rats

In the present study, Dulcolax S was used as the standard drug to treat constipated rats. As shown in Table 2, organ weights, namely weights of whole livers, kidneys, and hearts, were not affected by treatment with T-X or Dulcolax S, which indicated that T-X had no side effects *in vivo*.

**Table 2.** Effect of T-X treatment on organ weight in loperamide-induced constipated rats

	Groups			
	NOR	CON	T-X	DS
Liver	8.83 ± 0.16^NS^	8.38 ± 0.51	8.51 ± 0.36	8.26 ± 0.56
Kidney	2.18 ± 0.07^NS^	1.96 ± 0.11	1.86 ± 0.17	1.87 ± 0.18
Heart	0.99 ± 0.04^NS^	0.93 ± 0.03	0.94 ± 0.11	0.89 ± 0.03

NOR: normal diet group, CON: loperamide-induced constipation group (4 mg/kg, p.o.), T-X: taurine-xylose (15 mg/kg, p.o.) and loperamide-treated group, DS: Dulcolax S (5.5 mg/kg, p.o.) and loperamide-treated group. Values are the mean ± SD (n = 6). NS: not significantly different among groups.

### Effects of T-X on fecal parameters in rats with loperamide-induced constipation

**Figure 2.**
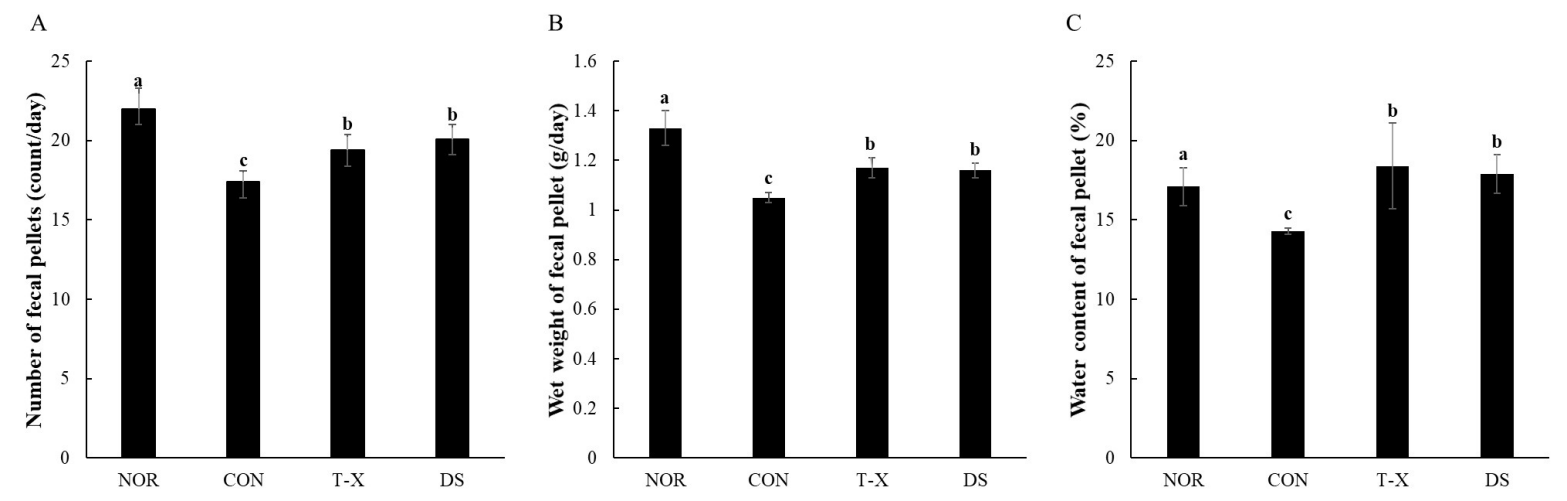
Effects of T-X on the number, weight, and water content of fecal pellets in loperamide-induced constipated rats. NOR: normal diet group, CON: loperamide-induced constipation group (4 mg/kg, p.o.), T-X: taurine-xylose (15 mg/kg, p.o.) and loperamide-treated group, DS: Dulcolax S (5.5 mg/kg, p.o.) and loperamide-treated group. Values are the mean ± SD (n = 6). Values not sharing a common letter are significantly different at P<0.05 by the Tukey-Kramer multiple comparison test.

The effects of T-X on the number, wet weight, and water content of fecal pellets from loperamide-induced constipated rats are shown in Fig. 2. To confirm that constipation was induced by loperamide, we investigated fecal parameters. In the CON group, the number of fecal pellets was significantly lower than that in the NOR group (17.4 counts/day in CON vs. 22.0 count/day in NOR), which indicated that constipation was establishment in response to loperamide. After constipation was induced, the number, wet weight, and water content of fecal pellets in the T-X and DS groups markedly increased relative to those in the CON group. In particular, T-X treatment increased the water content of fecal pellets above normal (18.4% in T-X vs. 17.1% in NOR).

### Effects of T-X on biochemical parameters in loperamide-induced constipated rats

To examine the effect of T-X on the regulation of biochemical parameters in loperamide-induced constipated rats, serum AST and ALT levels were determined to assess hepatic function and BUN levels were determined to assess renal function. As shown in Table 3, serum protein, albumin, BUN, and creatinine levels were not influenced by T-X treatment. In addition, indexes of liver cell injury, such as AST and ALT levels, were not significantly different among the groups.

**Table 3.** Effects of T-X treatment on serum biomarkers in loperamide-induced constipated rats

	Groups			
	NOR	CON	T-X	DS
Protein (g/dL)	6.43 ± 0.25^NS^	6.81 ± 0.46	6.72 ± 0.15	6.59 ± 0.30
Albumin (g/dL)	4.11 ± 0.27 ^NS^	4.03 ± 0.18	4.15 ± 0.31	4.23 ± 0.12
AST (U/L)	91.5 ± 5.2^NS^	102.8 ± 9.8	89.6 ± 10.2	97.3 ± 11.9
ALT (U/L)	40.8 ± 10.7^NS^	57.2 ± 19.6	39.2 ± 7.4	41.6 ± 8.8
BUN (mg/dL)	13.2 ± 3.0 ^NS^	14.1 ± 2.8	11.9 ± 2.6	12.8 ± 1.5
Creatinine (mg/dL)	0.40 ± 0.06^NS^	0.38 ± 0.10	0.39 ± 0.08	0.41 ± 0.09

NOR: normal diet group, CON: loperamide-induced constipation group (4 mg/kg, p.o.), T-X: taurine-xylose (15 mg/kg, p.o.) and loperamide-treated group, DS: Dulcolax S (5.5 mg/kg, p.o.) and loperamide-treated group. Values are the mean ± SD (n = 6). NS: not significantly different among groups. AST, aspartate aminotransferase; ALT, alanine aminotransferase; BUN, blood urea nitrogen.

### Hepatic GSH content and antioxidant enzyme activities

**Figure 3.**
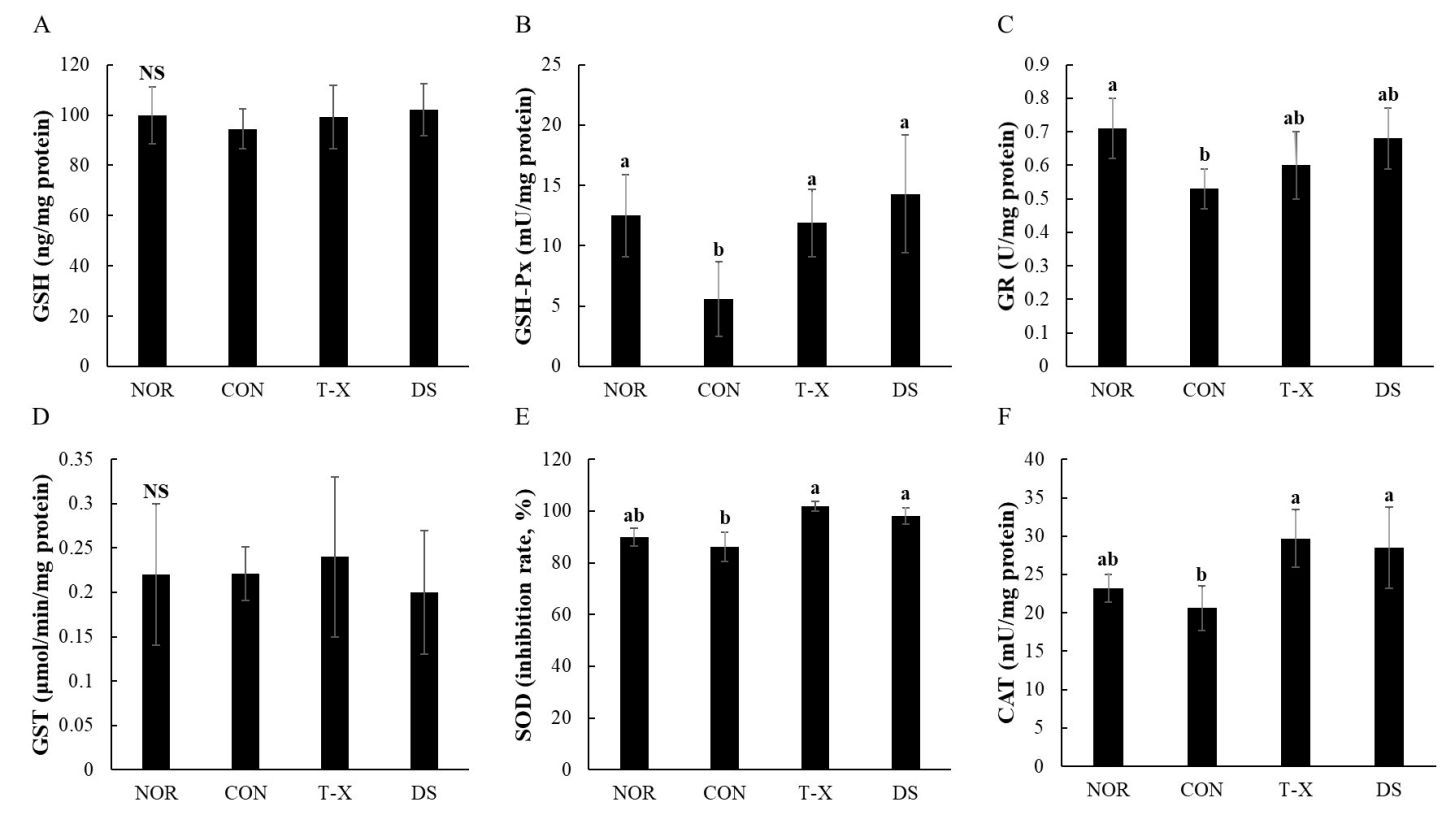
Effects of T-X treatment on hepatic GSH content, and antioxidant enzyme activities in loperamide-induced constipated rats. NOR: normal diet group, CON: loperamide-induced constipation group (4 mg/kg, p.o.), T-X: taurine-xylose (15 mg/kg, p.o.) and loperamide-treated group, DS: Dulcolax S (5.5 mg/kg, p.o.) and loperamide-treated group. Values are the mean ± SD (n = 6). NS: not significantly different among groups. Values not sharing a common letter are significantly different at P<0.05 by the Tukey-Kramer multiple comparison test.

Figure 3 shows the hepatic GSH content and antioxidant enzyme activities of rats with constipation induced by loperamide. In the present study, there were no significant differences in GSH content and GST activities among the groups. GR activity tended to increase in the T-X and DS groups, respectively, compared to that in the CON group, although there was no significant difference among the groups. In the CON group, antioxidant enzyme activities such as GSH-Px, GR, SOD, and CAT were markedly decreased compared to those in the NOR group, whereas GSH-Px, SOD, and CAT activities were significantly elevated in both the T-X and DS groups when compared with the CON group.

### Effect of T-X on gastrointestinal motility in rats with loperamide-induced constipation

**Figure 4.**
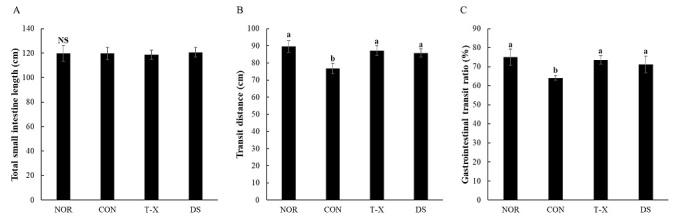
Effects of T-X treatment on gastrointestinal transit ratio in loperamide-induced constipated rats. NOR: normal diet group, CON: loperamide-induced constipation group (4 mg/kg, p.o.), T-X: taurine-xylose (15 mg/kg, p.o.) and loperamide-treated group, DS: Dulcolax S (5.5 mg/kg, p.o.) and loperamide-treated group. Values are the mean ± SD (n = 6). NS: not significantly different among groups. Values not sharing a common letter are significantly different at P<0.05 by the Tukey-Kramer multiple comparison test.

In the present study, we investigated the effects of T-X on gastrointestinal motility, as shown in Fig. 4. Indexes of gastrointestinal motility such as transit distance and GIT ratio markedly decreased in the loperamide-induced constipated rats. In contrast, treatment with T-X or Dulcolax S markedly increased the transit distance and GIT ratio in loperamide-induced constipated rats relative to those in the NOR group. These results indicated that T-X ameliorated constipation.

### Effects of T-X on serum gastrointestinal hormones in rats with loperamide-induced constipation

**Figure 5.**
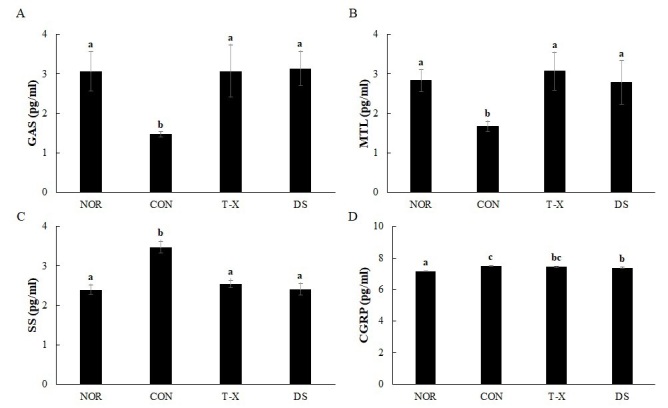
Effects of T-X on serum gastrointestinal hormones in loperamide-induced constipated rats. NOR: normal diet group, CON: loperamide-induced constipation group (4 mg/kg, p.o.), T-X: taurine-xylose (15 mg/kg, p.o.) and loperamide-treated group, DS: Dulcolax S (5.5 mg/kg, p.o.) and loperamide-treated group. Values not sharing a common letter are significantly different at P<0.05 by the Tukey-Kramer multiple comparison test. The concentrations of (A) GAS, (B) MTL, (C) SS, and (D) CGRP in the serum were estimated by ELISA.

We also evaluated the effects of T-X on serum gastrointestinal hormones, namely GAS, MTL, SS, and CGRP. As shown in Fig. 5, serum GAS and MTL concentrations in the CON group were significantly lower than those in the NOR group. However, colonic motility indexes such as serum GAS and MTL concentrations were markedly increased in the T-X and DS groups relative to those in the CON group. On the other hand, serum SS concentrations in the CON group were significantly higher than those in the NOR, T-X, and DS groups. Serum CGRP concentration was markedly higher in the CON group than in the NOR and DS groups; however, T-X did not change serum CGRP concentrations in loperamide-induced constipated rats.

## DISCUSSION

Digestive disturbances and irregularities in secretion or absorption caused by loperamide lead to oxidative stress in the intestines^23^. Free radicals are substances produced during cell metabolism, and these are toxic to several organs and tissues and can cause DNA, lipid, cell membrane, and protein damage^24^. In addition, reactive oxygen species (ROS) induce membrane lipid peroxidation, which damages cells, and play an important role in gastrointestinal diseases^25^. In the present study, we studied the antioxidant activities and therapeutic effects of T-X, a taurine-carbohydrate derivative on rats with loperamide-induced constipation. We found that T-X exhibited potent scavenger activities against DPPH and ABTS, as well as reducing power, *in vitro*. Similarly, taurine showed significant scavenging activities against DPPH and alkyl radicals^26^.

Constipation is a common gastrointestinal disorder. It is characterized by symptoms such as poor appetite, infrequent bowel movements, a bloated abdomen, and difficulty defecating. In addition, toxins in the stool that have not been released are absorbed into the intestines and can cause various diseases^27^. Loperamide is commonly used to induce constipation in animal models. It can slow the movement of stool and extend the stool drain period^28^. In the present study, we investigated the laxative effects of T-X in rats with constipation induced by loperamide. We found that final body weight and body weight gain were significantly increased in the NOR group relative to those in the other groups. Food intake tended to decrease in the CON group compared to that in the T-X and DS groups. However, there was no significant difference in FER and organ weights among the groups. These results indicated that neither T-X and Dulcolax S altered FER, organ weight, or food intake in constipated rats.

Numerous studies have shown that a significant marker of constipation in loperamide-induced rats is a reduction in fecal excretion^29,30^. In this study, fecal parameters such as number, wet weight, and water content of fecal pellets in the CON group were significantly decreased relative to those in the NOR group. Conversely, these fecal parameters were significantly increased by T-X treatment. These results suggest that T-X treatment had a protective effect against loperamide-induced constipation. Similarly, Kim et al.^31^ reported that taurine-galactose supplementation markedly increased fecal parameters, including the number and weight of fecal pellets, in loperamide-treated rats with constipation.

In normal conditions, the activities of intracellular enzymes such as ALT and AST remain low in serum. When hepatocytes are damaged, however, their cell membrane permeability increases, resulting in an increase in their levels in serum. Therefore, serum ALT and AST levels are commonly used as indicators of hepatic function^32^. In the present study, T-X treatment did not affect serum biomarkers, including protein, albumin, AST, ALT, BUN, and creatinine levels. These results suggested that T-X treatment did not alter hepatic and renal function in loperamide-induced constipated rats.

On another hand, ROS, particularly hydroxyls radicals, can induce lipid peroxidation of cell membranes, causing significant cellular damage and leading to gastrointestinal diseases^33^. In recent previous studies, a correlation between oxidative stress and pathogenesis in loperamide-induced constipated rats was established^33,34^. In our study, we confirmed that hepatic antioxidant enzyme activities such as those of GSH-Px, SOD, and CAT were markedly increased in both T-X- and DS-treated rats compared to those in the CON group. In a previous study, we confirmed that xylose-taurine-reduced (X-T-R), a taurine derivate, improved the survival rate of zebrafish embryos by suppressing intracellular NO and ROS generation^35^. X-T-R also showed potent hepatoprotective effects against oxidative damage induced by hydrogen peroxide in response to Nrf2 signaling^36^. Moreover, SOD content was reported to decrease and MDA content reported to increase in both serum and the intestines in constipated rats, indicating that constipation may cause oxidative products to accumulate and antioxidative defenses to be weakened^37^. Our findings suggest that T-X treatment can help alleviate oxidative stress in loperamide-induced constipated rats.

In our study, carmine was used as a marker to evaluate the GIT ratio in loperamide-induced constipated rats. GIT is known to reflect total gastrointestinal activity^38^. In the present study, T-X treatment significantly increased the transit distance and GIT ratio compared to those in the CON group. Similarly, our previous findings have indicated that taurine supplementation markedly and dose-dependently increases the GIT ratio in loperamide-induced constipated rats^39^.

In general, digestive juice secretion, movement of gastrointestinal content, and gastrointestinal motility are known to be stimulated by gastrointestinal hormones such as GAS and MTT and inhibited by SS and CGRP^40^. In the present study, serum concentrations of GAS and MTL were significantly increased and serum SS concentrations decreased by T-X treatment of loperamide-induced rats. These results indicate that T-X can be effective and useful as a laxative in improving colonic motility.

In conclusion, our results show that T-X treatment enhanced hepatic antioxidant enzyme activities and improved several fecal parameters, the GIT ratio, and serum gastrointestinal hormone levels in loperamide-induced constipated rats. However, our findings were limited in that our constipation was only induced by loperamide in our animal model. Therefore, further studies are necessary to clarify the laxative effects and mechanisms of action of T-X in humans.
